# Three-dimensional rocking curve imaging to measure the effective distortion in the neighbourhood of a defect within a crystal: an ice example

**DOI:** 10.1107/S002188981300472X

**Published:** 2013-06-07

**Authors:** Armelle Philip, Jacques Meyssonnier, Rafael T. Kluender, José Baruchel

**Affiliations:** a UJF-Grenoble 1/CNRS, Laboratoire de Glaciologie et Géophysique de l’Environnement (LGGE), UMR 5183, BP 53, Grenoble, F-38041, France; b European Synchrotron Radiation Facility, 6 rue Jules Horowitz, BP 220, Grenoble, F-38043, France

**Keywords:** rocking curve imaging (RCI), 3D-RCI, ice crystals, crystal distortion, crystal defects

## Abstract

Rocking curve imaging (RCI) is a quantitative version of monochromatic beam diffraction topography that involves using a two-dimensional detector, each pixel of which records its own ‘local’ rocking curve. From these local rocking curves one can reconstruct maps of particularly relevant quantities (*e.g.* integrated intensity, angular position of the centre of gravity, FWHM). Up to now RCI images have been exploited in the reflection case, giving a quantitative picture of the features present in a several-micrometre-thick subsurface layer. Recently, a three-dimensional Bragg diffraction imaging technique, which combines RCI with ‘pinhole’ and ‘section’ diffraction topography in the transmission case, was implemented. It allows three-dimensional images of defects to be obtained and measurement of three-dimensional distortions within a 50 × 50 × 50 µm elementary volume inside the crystal with angular misorientations down to 10^−5^–10^−6^ rad. In the present paper, this three-dimensional-RCI (3D-RCI) technique is used to study one of the grains of a three-grained ice polycrystal. The inception of the deformation process is followed by reconstructing virtual slices in the crystal bulk. 3D-RCI capabilities allow the effective distortion in the bulk of the crystal to be investigated, and the predictions of diffraction theories to be checked, well beyond what has been possible up to now.

## Abstract 2

A three-dimensional Bragg diffraction imaging technique, which combines rocking curve imaging with ‘pinhole’ and ‘section’ diffraction topography in the transmission case, allows three-dimensional lattice distortion in the bulk of an ice crystal under compression to be measured.

## 1. Introduction

Rocking curve imaging (RCI), which has developed over recent years at modern synchrotron radiation facilities (Lübbert *et al.*, 2000 ▶; Mikulik *et al.*, 2006 ▶), is a directly quantitative version of monochromatic beam diffraction topography. RCI involves using a two-dimensional detector (CCD camera) to record the diffracted spot. Each pixel of the camera records its own ‘local’ rocking curve (RC), so that several images (or ‘maps’) can be reconstructed by extracting data from these local RCs. Three of these maps are particularly relevant: they display the integrated intensity, the angular position of the centre of gravity and the full width at half-maximum (FWHM) of the local rocking curves. Up to now these RCI images were exploited in the reflection case, providing a quantitative picture of the features present in a several-micrometre-thick subsurface layer.

A three-dimensional Bragg-diffraction imaging technique (3D-RCI), which combines RCI with ‘pinhole’ and ‘section’ diffraction topography in the transmission case, has been successfully implemented (Kluender *et al.*, 2011 ▶; Kluender, 2011 ▶). It allows three-dimensional distortions within a 50 × 50 × 50 µm elementary volume (a ‘voxel’) and angular misorientations down to 10^−5^–10^−6^ rad to be measured and three-dimensional images of defects to be obtained. This technique was initially designed with the aim of obtaining the three-dimensional curvature tensor of a sample: indeed computing the lattice curvature from lattice orientation measurements, in particular near grain boundaries, and relating this curvature to the presence of a density of ‘geometrically necessary dislocations’, provides a promising experimental approach for the study of plasticity in crystalline materials (Nye, 1953 ▶; Sun *et al.*, 2000 ▶).

The viscoplastic deformation of crystalline materials occurs by dislocation glide on preferential crystallographic planes. The fact that some slip systems are privileged in a single crystal can lead to an anisotropy of its mechanical behaviour. When a polycrystal deforms, strain incompatibilities arise at grain boundaries: each grain cannot deform in isolation because its neighbouring grains constitute an additional constraint. These incompatibilities show up in particular at grain boundaries and generate stress concentrations that provoke intragranular strain heterogeneity. Studying strain incompatibility at a grain boundary and intragranular strain heterogeneity is very important to explain strain hardening and fracture processes in polycrystalline materials, which start at a scale that is smaller than the grain size.

The aim of the present paper is neither to explain the details of the 3D-RCI technique, which have already been presented (Kluender *et al.*, 2011 ▶; Kluender, 2011 ▶), nor to show how the recorded curvature tensors can be used to give clues on anisotropic plasticity. The present paper topic, after a very brief description of the experimental techniques, is to present the many local quantitative results, which are well beyond what have been measured up to now, on the deformation in the neighbourhood of bulk defects.

These results were obtained using 3D-RCI to follow the inception of the deformation process of one of the grains of a three-grained ice polycrystal. They are extracted from the above-mentioned maps of the integrated intensity, peak position and width of the local rocking curves for virtual slices located in the bulk of the ice crystal. This experimental approach allows investigation of a region within the volume of the crystal, without perturbation by surface effects. More particularly, we will discuss results on the inner distortion of an ice voxel containing a dislocation, the peak position of the corresponding rocking curve, the way this evolves when glide planes are activated and the initial polygonization state that appears when deforming the crystal.

## 2. Sample and experimental techniques

A three-grained polycrystal, *i.e.* a tricrystal, which exhibits grain boundaries and a triple junction, is the simplest model for a polycrystalline material. On the other hand, ice presents a series of advantages for an investigation of the type presented here: X-ray absorption by ice is very low, ice is hexagonal and deforms mainly by basal dislocation glide (Duval *et al.*, 1983 ▶), thus displaying a very strong viscoplastic anisotropy, and the mobility of dislocations is low (∼5 µm s^−1^ MPa^−1^ at 258 K; *e.g.* Hondoh, 2000 ▶), thus allowing the motion to be followed during the early stages of deformation.

**Figure 1:**
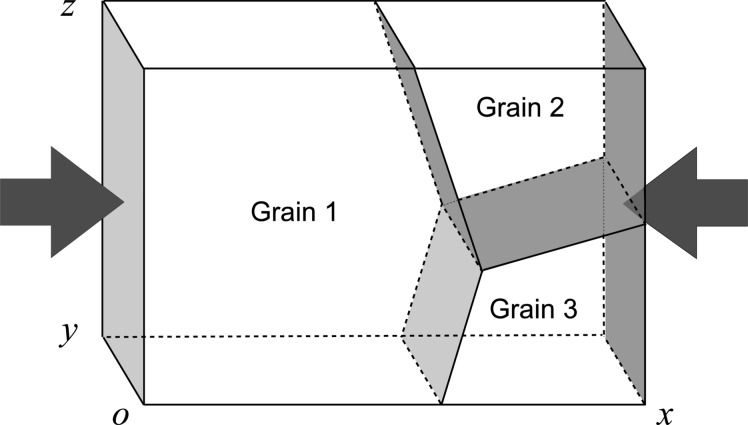
Schematic of the tricrystal sample. The *c* and *a* axes of Grain 1, expressed in the sample reference frame indicated on the figure, are parallel to the vectors (0.99, −0.11, 0.06) and (0.09, 0.89, 0.13), respectively. The arrows indicate the direction of compression (*x* axis). The sample is 21 mm long (*x* axis), 17 mm wide (*z* axis) and 4 mm thick (*y* axis).

All of these considerations led us to choose, for the present investigation, a tricrystal of ice with grains of a high crystalline quality to allow observation of the inception of plastic deformation (Fig. 1 ▶). A growth method for ice multi-crystals and a protocol for specimen preparation were worked out, resulting in ice grains with a low initial dislocation density (∼10^2^ cm cm^−3^) and large grains (∼80 mm) (Baruchel *et al.*, 2013 ▶).

The experimental conditions for 3D-RCI of the ice tricrystal are as follows: the ice sample is milled to a parallelepiped shape (21 × 17 × 4 mm) and placed in a plastic casing adjusted to fit the specimen dimensions to prevent its sublimation. The sample is placed in a double-walled cell and refrigerated at 263 K by a constant air flow coming from a cryostat. This environment, weakly absorbing for the 18 keV X-rays used in the present work, also allows the sample in compression to deform under plane strain conditions with the help of a pneumatic press. The cell was installed on a goniometer (diffractometer) on the ESRF beamline BM05, allowing the crystal to be oriented with respect to the X-ray beam in the appropriate crystallographic positions.

In usual projection X-ray diffraction topography, a three-dimensional sample is imaged on a two-dimensional detector. This implies that the whole sample volume is projected onto the same pixel of the detector, which could lead to the superposition of defect images. The 3D-RCI method we developed overcomes this limitation by combining a series of monochromatic beam section topographs and RCI in order to obtain a mapping of the complete crystalline orientation, in voxels of 50 µm size, within a volume of about 500 mm^3^ (Kluender *et al.*, 2011 ▶; Kluender, 2011 ▶).

Section topography (Lang, 1957 ▶; Andersen & Gerward, 1974 ▶) is a straightforward way to access detailed volume information (Kawado & Aoyama, 1979 ▶; Kvardakov *et al.*, 2007 ▶; Allen *et al.*, 2011 ▶), because only a section of the crystal is illuminated, corresponding to the volume limited by the slit size and the sample thickness. In spite of the very high initial quality of the crystal, interference fringes (‘Kato’s fringes’; see for instance Authier, 2001 ▶) are only observed in a very few of the recorded section images. To a good approximation, we can consider that the borders of the section image on the detector correspond to the two crystal surfaces and the inside image corresponds to the crystal bulk illuminated by the incident planar beam. The multi-slit device we used is based on an absorbing gold layer deposited on a silicon plate, which allows the transmission of 20 parallel micro-beams spaced 1 mm apart, each of them being 15 mm wide and 50 µm thick. In order to manufacture the slits, thin gold wires perpendicular to the slits were required: they appear as thin ‘absorption lines’, independent of the features of the sample, on the recorded images. The multi-slit device is displaced 20 times by 50 µm to scan the crystal through the entire size of the incident synchrotron X-ray beam. In practice at least 21 scans are recorded to get a superposition of the last one with the first. This *modus operandi* produces artefacts parallel to the slits, corresponding to the passage of the 20th slit to the first slit, which are associated with the displacement of the section setup during the scan: since the first and 20th data acquisitions are performed with an interval of several hours, the synchrotron radiation incident beam conditions can differ, and also, when analysing a compressed sample, although the sample was unloaded prior to scanning, a slight residual deformation can occur from the beginning to the end of the scan.

In the RCI technique, the sample is oriented in such a way that one chosen crystallographic plane is Bragg diffracting and is stepwise rocked by an angle Δω around a reference value ω_0_. At each crystal position, the Bragg diffracted beam RC is registered on a FReLoN-type two-dimensional CCD detector, equipped with a scintillator and visible light optics. The pixel size of the CCD camera is 10 µm. The final data are composed of a stack of RCI images, where each image corresponds to the diffracted beam intensity *I*(*y, z*, ω) for the sections provided by the multislit device for a crystal orientation ω and at each camera pixel (*y, z*). When taking into account the detector spatial resolution, the width of the slit and the geometrical projection effects, our spatial resolution corresponds to a voxel of 50 × 50 × 50 µm. By analysing all the local rocking curves (Gaussian shape in our study approximates each local RC), it is possible to draw, as indicated before, maps of the integrated intensity, of the angular position of the centre of gravity of the RC and of the FWHM. In our experiments the effect of the variation of the ice lattice parameters is very small with respect to the misorientations that occur within the crystal when deformed. The position of the centre of gravity therefore corresponds to the local crystalline orientation of the crystal volume producing the local RC. The angular resolution depends on the chosen step size and on the energy resolution of the monochromator: it is in the sub-µrad range. The FWHM map quantifies the inner distortion (‘mosaicity’) of the ice voxel producing the locally diffracted beam recorded on a given pixel for each of the sections.

## 3. Data acquisition

The experimental procedure described was applied to the study of the microstructure evolution of one grain of an ice tricrystal under planar compression (Grain 1 in Fig. 1 ▶). The time for data acquisition of all the experimental RCI data for a given plane is in the range 4–18 h. This time depends on the beam intensity for the detector exposure time, the angular step size used for the rocking acquisition and the quality of the analysed crystal.

Data acquisition was performed during three main steps: firstly, with the sample in its initial state and then after two 40 min compression sessions (under 1.1 MPa) along the longitudinal axis of the sample. For each of these steps the 3D-RCI procedure, which aims at measuring the complete three-dimensional curvature tensor and therefore having access to three independent angles for each voxel, consisted of measuring two reflections (the basal 0002 and prismatic 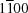) plus performing a series of pinhole topographs to measure the plane misorientation around the direction of the incident beam (Kluender *et al.*, 2011 ▶; Kluender, 2011 ▶). By stacking up the data from the RCI sections (in the direction of the diffraction vector) one obtains, for each analysed plane, a series of three-dimensional matrices (integrated intensity, angular position, FWHM). Numerically ‘cutting’ through such a matrix allows visualization of any desired virtual section inside the crystal.

The recorded rocking curves are slightly broadened by dispersive effects due to geometrical aspects related to the synchrotron X-ray source and the monochromator position, which broaden the rocking curve more in the horizontally diffracting geometry than in the vertical one. These dispersive effects, which are in particular relevant for the initial, not deformed, state, where the RCs are very narrow (∼µrad range), are calculated and removed in the following figures, in such a way that the discussion will not have to take this effect into account (Kluender, 2011 ▶).

## 4. Results and discussion

### 4.1. Initial, nondeformed, state

**Figure 2:**
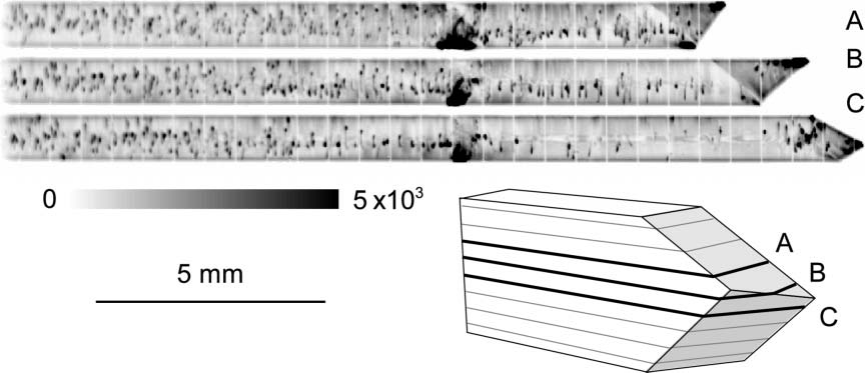
Integrated intensity for three horizontal sections (A, B, C) of the main tricrystal grain (Grain 1 in Fig. 1 ▶) in the initial state: prismatic plane vertically diffracted.

Let us first remark that the RCI-section reconstructed virtual slice images of the integrated intensity obey the known visibility criteria for defects established for usual section or projection topographs (Authier, 2001 ▶). In the initial state of the crystal, characterized by a very low dislocation density, individual dislocations can be observed on the integrated intensity RCI images. The basal section maps do not exhibit dislocation images. When observing the integrated intensities of the prismatic reflection, the intersections of dislocation lines with the crystal volume illuminated by the section beams appear as points in the sections, as shown in Fig. 2 ▶, and as lines parallel to the basal planes in the virtual slices parallel to the main surfaces. From these images, we can conclude that the Burgers vector of these dislocations lies in the basal plane. By following the ‘trajectory’ of one dislocation point in the series of section images along the direction normal to the prismatic plane we can derive the orientation of the associated dislocation line, which appears to be perpendicular to the 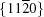 prismatic planes. This allows us to conclude that they are screw dislocations with, very probably, Burgers vector (1/3)〈
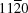
〉, *i.e.* the most observed ones in ice.

Under this assumption we can calculate the width *w* of the associated dislocation image as seen on the prismatic lattice planes. The direct image of a dislocation is predominant in the low absorption case we are concerned with. Its ‘intrinsic’ width *w*_i_ originates from a distorted region around the defect, where the induced lattice distortions exceed αω_D_, with ω_D_ the intrinsic width of diffraction for the perfect crystal and α a value in the range 0.5–1.5 (Authier, 2001 ▶; Miltat & Bowen, 1975 ▶; Klapper, 1976 ▶). The experimental dislocation image width results from the convolution of this intrinsic width *w*_i_, the detector resolution 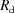 and the image widening associated with the angular divergence of the Bragg diffracted beam *L*, that is 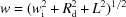. In our case *w*_i_ is 5 µm for the mentioned basal screw dislocations, visible in the prismatic image, the detector resolution is 20 µm and the divergence of the Bragg diffracted beam, given by the FWHM of the rocking curve in a dislocation region, is ∼2.5 µrad. As the distance between the sample and the detector was 1 m, the broadening due to the divergence of the Bragg diffracted beam is ∼50 µm, being therefore the dominant factor in the convolution and leading to *w* ≃ 54 µm, a value which is in keeping with the measured one.

**Figure 3:**
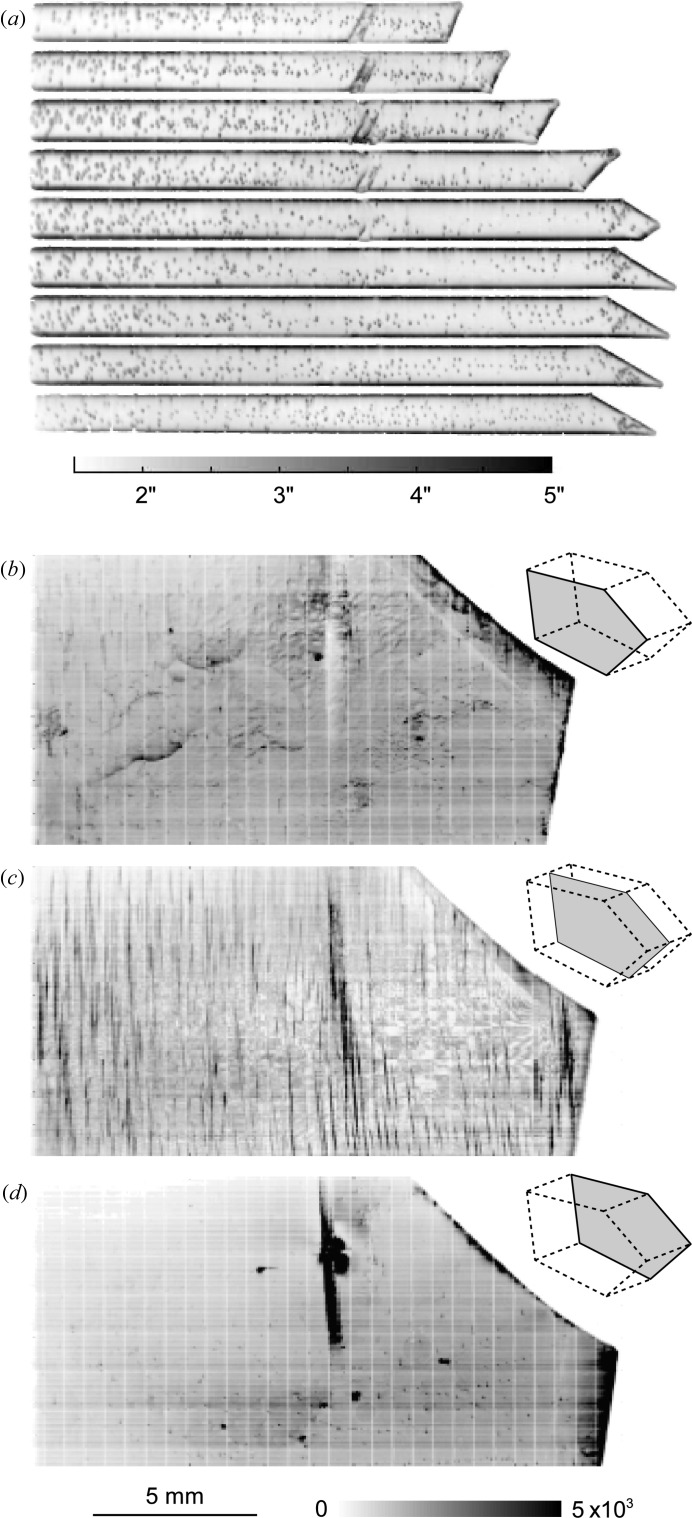
Two-dimensional maps showing the FWHM sections (*a*) and the integrated intensities in the central (*c*) and two surface (*b*), (*d*) reconstructed virtual slices of the main tricrystal grain (Grain 1 in Fig. 1 ▶) in the initial state: prismatic plane vertically diffracted.

On the FWHM RCI-section virtual slice images we observe very similar features to the ones described above for integrated intensity images. The dislocation images are practically not visible on the FWHM maps of the basal reflection, where most of the pixels display the intrinsic width of diffraction (*i.e.* Darwin width) foreseen by the dynamical theory of diffraction (see for instance Authier, 2001 ▶). Fig. 3 ▶(*a*) shows the recorded FWHM of the prismatic reflection section images, before the three-dimensional integration, and Figs. 3 ▶(*b*), 3 ▶(*c*) and 3 ▶(*d*) the integrated intensity for the central and surfaces virtual slices, reconstructed from all the section images. The central slice, 50 µm thick, is located in the middle of the 4 mm-thick crystal and cuts the triple junction line where the three grains meet. This junction plays a very important role in the deformation process, as pointed out later. As for the basal plane reflection, most of the pixels that do not display a dislocation exhibit an FWHM of the order of the intrinsic width of diffraction. This is not true for the surfaces of the crystal, which display a higher diffracted intensity and FWHM of the RC owing to the distortions introduced by the sample manufacturing process.

The presence of one dislocation in the otherwise perfect crystal was found to increase the FWHM of the prismatic reflection rocking curve of the associated ice voxel to a value of about 3–4′′, about twice that of the perfect crystal matrix [see Fig. 3 ▶(*a*) and Fig. 7(*a*) below]. This is in agreement with a direct calculation of the average mosaic spread of the prismatic planes seen, over a 50 µm path, by a 10 µm-wide X-ray beam travelling perpendicular to a screw dislocation lying in the basal plane (10 µm corresponds to the CCD camera pixel size). On the other hand, whereas the global dislocation density in the crystal (∼4 × 10^2^ cm cm^−3^) is too low to broaden the RC of the entire Bragg diffraction image (Kaganer & Sabelfeld, 2010 ▶), this density, when referred to a voxel (50 × 50 × 10 µm) containing a single dislocation, would correspond to a value of 2 × 10^5^ cm cm^−3^.

**Figure 4:**
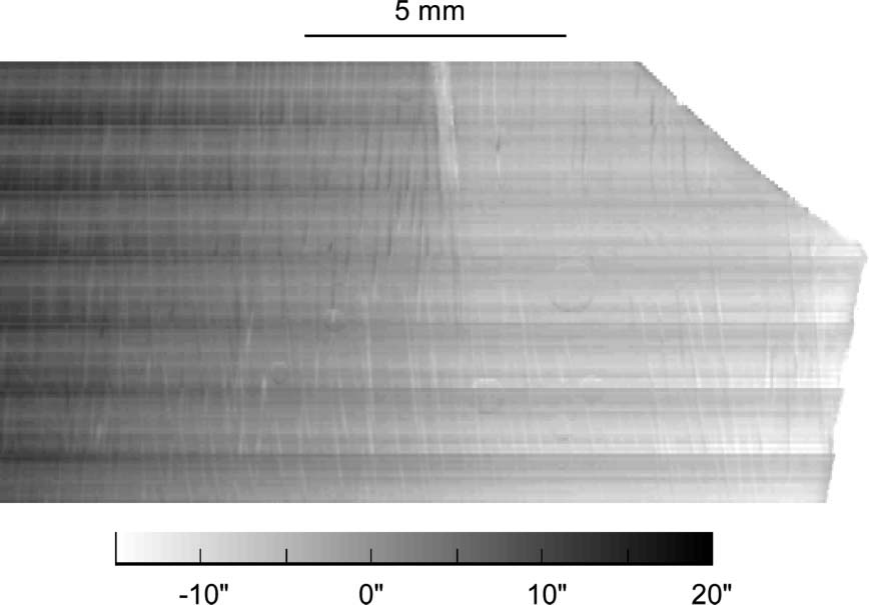
Angular RC peak shift with respect to the nominal Bragg angle in the central reconstructed virtual slice of the main tricrystal grain (Grain 1 in Fig. 1 ▶) in the initial state: prismatic plane vertically diffracted.

**Figure 5:**
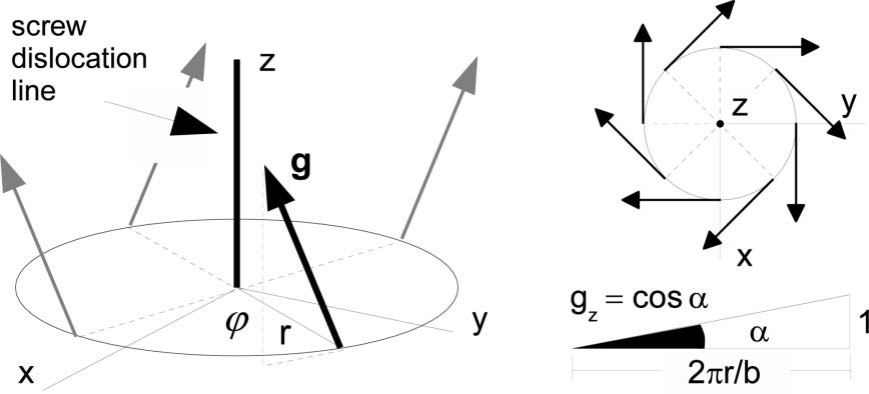
Shape of the normal vector field of the prismatic lattice planes associated with a basal screw dislocation.

More surprisingly, dislocation images are also visible on the maps of the angular position of the RC centre of gravity (Fig. 4 ▶), where they shift the RC peak by 2′′ in the negative or positive direction. The origin of this shift does not reside in a misorientation associated with the presence of the screw dislocation, because the average misorientation of the prismatic planes in a voxel containing a basal screw dislocation is zero, the vector field of the normals to the prismatic planes in the surrounding area of a basal screw dislocation exhibiting a circular symmetry. The vector normal to the prismatic lattice planes 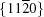 is parallel to the basal screw dislocation line far from the dislocation core and increasingly inclines the closer we get to the dislocation core. Yet, on a circle around the dislocation core, the angle between the normal vector of the lattice planes and the dislocation line is a constant (Fig. 5 ▶). As a result, the misorientation of the lattice planes on one side of the dislocation is cancelled out by the one on the opposite side: the average orientation of the prismatic lattice planes in a voxel is, therefore, not affected by the presence of a basal screw dislocation.

However, if the dislocation line is not centred in the voxel but closer to its edge, there may be a net contribution of the distortion field and a shift in the mean orientation of the voxel. This mechanism would lead to the visibility of (very roughly) about half the dislocations, *i.e.* the ones located near the edges of the voxel. Comparison of the integrated intensity map with the FWHM shows that nearly all dislocations are visible on this last image. Another feature could explain the shift of the RC peak in the dislocation region: an incident beam that propagates through a dislocation line crosses one lattice plane less (or one lattice plane more, depending on the orientation of the Burgers vector of the screw dislocation). The thickness of a virtual slice is approximately 50 µm while one lattice spacing distance is ∼5 × 10^−4^ µm. This leads to a variation in the effective lattice spacing due to the presence of the screw dislocation Δ*d*/*d* ≃ 10^−5^. The variations in the effective lattice spacing are associated with a difference in the Bragg angle and an angular shift Δθ = (Δ*d*/*d*)tanθ, visible on the RC peak position maps. The Δθ difference in the Bragg angle resulting from such a mechanism appears to be in keeping with the values that we have measured.

**Figure 6:**
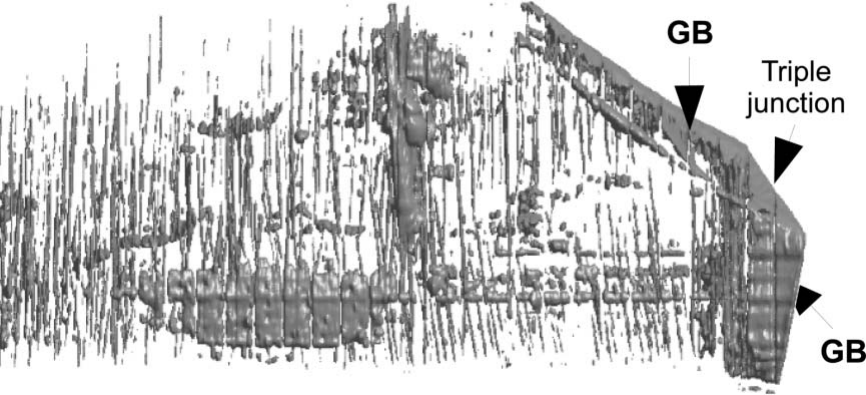
Three-dimensional reconstruction of the dislocations in the main tricrystal grain (Grain 1 in Fig. 1 ▶) in the initial state.

From the RCI stack of sections it is of course possible to extract a three-dimensional rendering of the dislocations present in the initial state of the grain we are concerned with (Fig. 6 ▶).

### 4.2. Deformed states

Two steps of deformation were obtained through two successive applications of an external compressive stress of 1.1 MPa over a period of 40 min. The RCI experiments were performed, in both cases, 1 h after unloading the specimen.

**Figure 7:**
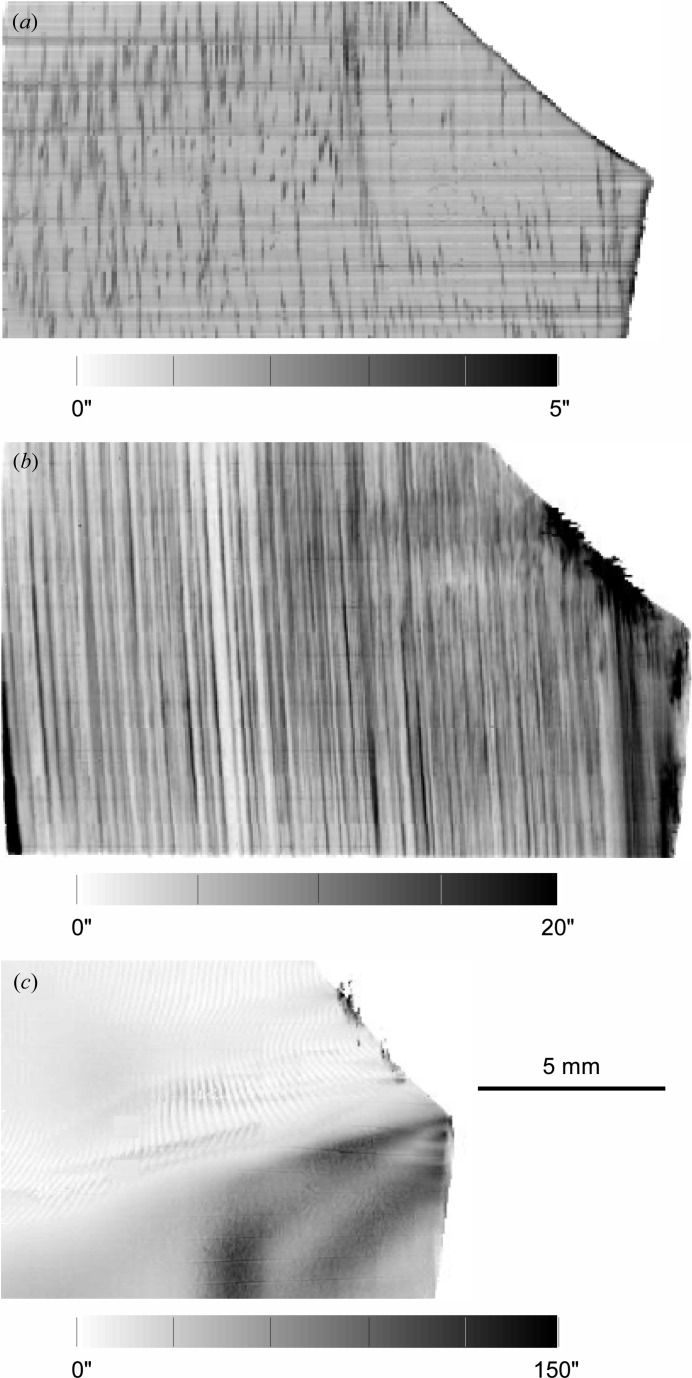
FWHM maps of the central virtual slice in (*a*) the initial state (prismatic plane), (*b*) the first step of deformation (prismatic plane) and (*c*) the final state (basal plane).

Fig. 7 ▶ shows, for comparison, the FWHM maps of the central virtual slice in (*a*) the initial state, with individual dislocation images, (*b*) the first step of deformation, with the occurrence of slip bands, which exhibit FWHM values two to three times higher than the voxels containing individual dislocations, and (*c*) the final state, after a further application of the external stress, which shows the inception of the formation of a subgrain boundary, in the neighbourhood of the triple junction, with FWHMs two orders of magnitude higher than for dislocations.

In the first step of deformation, the stress application induced a broadening of the global RC. The basal RC was recorded in an angular interval Δω = 0.13° and the prismatic one in Δω = 0.09°, which is approximately ten times higher than in the initial state. At this point the dispersive effects are negligible; the broadening of the RC arises from crystal lattice distortions due to the viscoplastic deformation. The basal rocking curve still has a one-peak shape, whereas the prismatic one reveals the first polygonization effects: it contains a main peak characterized by an FWHM of about 0.005° and a background where at least two broader peaks can be distinguished.

Already, after the first loading of the crystal, the basal screw dislocation density seen on prismatic images is too high to resolve single dislocations (Fig. 7 ▶ *b*). The dislocations are arranged into basal glide planes which allow the plastic deformation of the crystal. The glide planes are visible as parallel lines on the prismatic integrated intensity and FWHM images. On these last images (Fig. 7 ▶ *b*) the glide planes exhibit values in the range 5–20′′. In the initial state the presence of one dislocation in the prismatic image is associated with an additional broadening of the local RC peak which goes from 1.5–2′′ to 3–4′′. If we consider, as a first approximation, a linear dependency between the FWHM of the voxel rocking curve and the number of basal dislocations in that elementary volume, we can associate an FWHM value of 20′′ in a glide plane with the presence, within this volume, of about five to seven dislocations.

The second loading (Fig. 7 ▶ *c*) led to a further broadening of the global RC. The basal RC was recorded over an angular interval Δω = 0.5° where polygonization is visible through the occurrence of several peaks and the prismatic one over Δω = 0.8°, with tails originating from regions close to the triple junction. These angular widths are five to ten times higher than for the previous deformed state: they reveal the distortion of the ice lattice, which can be roughly summarized as follows. At the very beginning of the deformation the number of activated basal planes (the preferential glide planes) increases. However, because the three grains of the sample exhibit different, thus incompatible, basal orientations, the deformation becomes heterogeneous, particularly close to the grain boundaries and the triple junction. To accommodate this heterogeneity, the ice crystal lattice must undergo a distortion, which increases until the formation of a sub-boundary relaxes the amount of stored energy.

Let us note that the oscillatory features that can be observed in the upper part of Fig. 7 ▶(*c*) are artefacts that occur in RCI when the angular steps that are made along the RC are too large. The artefact disappears when the step size is reduced. The reason why the number of points of the RC was not high enough is that the overall deformation led us to scan, as indicated above, over an angular interval of 0.8°. The number of points per RC should have been increased by at least a factor of three to eliminate the artefact, implying a scan time three times longer, *i.e.* 24 h instead of 8 h. This was not compatible with the allocated experimental time, and therefore this difficulty might be regarded as a technical limit of our experimental procedure in terms of the observable crystal quality. The technique is perfectly suited for the early stages of plastic deformation where the crystal distortions are small and a high angular resolution is needed. For a deformation as was reached after the second loading of the crystal, techniques with lower angular resolution that can measure over larger angular intervals, as described by King *et al.* (2008 ▶), offer a solution.

**Figure 8:**
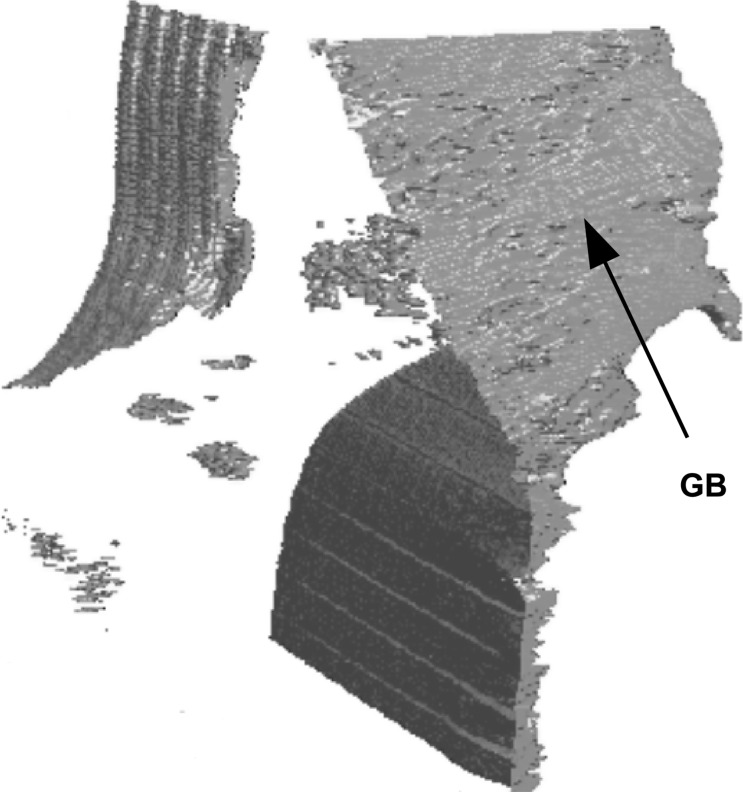
Three-dimensional reconstruction of the formation of a subgrain boundary, linked to the triple junction (GB), in the final state.

In spite of this limitation, the virtual slices allow a quantitative measurement of the local distortion in the course of this process: the FWHM values on the basal image of the regions located above/below the triple junction area exhibit values of ∼30′′, whereas, starting from the triple junction, a region constituting the inception of a subgrain boundary exhibits values up to 120′′. This forming subgrain boundary, linked to the triple junction, is a three-dimensional feature, as indicated in Fig. 8 ▶ which shows the three-dimensional rendering obtained from the RCI matrix. This confirms that the deformation mechanism of ice is an actual three-dimensional phenomenon, and in order to understand this process, three-dimensional data are required. Movie 1 in the supplementary material^1^ shows a three-dimensional reconstruction of the formation of a subgrain boundary associated with the triple junction, in the final state of deformation (see Fig. 8 ▶), and Movie 2 shows the FWHM three-dimensional map in the initial state.

## 5. Conclusion

Experimental results obtained with the new 3D-RCI technique, which combines rocking curve imaging with pinhole and section diffraction topography in the transmission case, have been presented. To discuss the capabilities of this technique, the inception of the plastic deformation inside one grain of a tricrystal of ice has been analysed by reconstructing three-dimensional data (integrated intensity, angular position of the centre of gravity and FWHM) from the local rocking curves recorded on every pixel of the CCD detector.

The 3D-RCI technique proves that three-dimensional images of the defects can be obtained and three-dimensional distortions within 50 × 50 × 50 µm elementary volumes in the bulk of the crystal with angular misorientations as low as ∼10^−5^–10^−6^ rad can be measured. As well as giving access to the effective distortion in the bulk of a crystal, this technique also allows predictions of diffraction theories to be checked, well beyond what has been possible up to now. The values of integrated intensities and FWHM in the neighbourhood of the defects within the bulk of the crystal, as well as the surprising visibility of some of these defects in the rocking curve angular misorientation images, and the quantification of the distortions associated with glide planes and the inception of a subgrain boundary, are original results, which can only be observed through the described procedure.

Although the gain of spatial resolution stands against a strongly increased experimental time (recording the RCI images on various angular orientations of the crystal with the multi-section device implies scanning times which are more hours than minutes), the results presented here show that 3D-RCI reveals much additional information that would not be accessible to the scientist otherwise. We could, in this way, obtain quantitative data on the initial stages of ice deformation that cover two orders of magnitude. Indeed, in the initial state, we have measured widths of the local rocking curves of about 1–2′′. This distortion is increased by one order of magnitude in the intermediate state at the level of the slip planes. In the final state, we have measured more than 200′′ in the neighbourhood of the triple junction, over the extended region that will produce a subgrain boundary.

These results provide unique information which shows that the 3D-RCI technique is an invaluable tool for studying the initial stages of the deformation process of a crystal.

## Supplementary Material

Movie 1. DOI: 10.1107/S002188981300472X/xk5009sup1.avi

Movie 2. DOI: 10.1107/S002188981300472X/xk5009sup2.avi

Supplementary material file. DOI: 10.1107/S002188981300472X/xk5009sup3.pdf

## Footnotes

1 Movies S1 and S2 for this paper are available from the IUCr electronic archives (Reference: XK5009). Services for accessing these materials are described at the back of the journal.
