# Supplementary material for: Three-dimensional rocking curve imaging to measure the effective distortion in the neighbourhood of a defect within a crystal: an ice example
Source: J Appl Crystallogr. 2013 Jun 7;46(Pt 4):842–8. doi: 10.1107/S002188981300472X (PMC3769054; doi:10.1107/S002188981300472X)
Supplement: Supplementary file 3 [file j-46-00842-sup3.pdf]

Video 1 corresponds to Figure 8, showing the 3D reconstruction of the formation of a subgrain boundary associated to the triple junction, in the final state of deformation.

Video 2 shows the FWHM 3D map in the initial state, reconstructed from sections as those shown in Figure 3a. The video starts from one surface of the crystal to the other. Except for the two surface layers the dislocations are clearly visible in the bulk of the crystal. The frames are spaced 50μm apart.
